# Genome-Wide Association Study of Birth Wool Length, Birth Weight, and Head Color in Chinese Tan Sheep Through Whole-Genome Re-Sequencing

**DOI:** 10.3390/ani14233495

**Published:** 2024-12-03

**Authors:** Lina Ma, Wei Zhao, Qing Ma, Jin Wang, Zhengwei Zhao, Juan Zhang, Yaling Gu

**Affiliations:** 1 College of Animal Science and Technology, Ningxia University, Yinchuan 750021, China; malina_2007nian@163.com (L.M.); z1729921403w@163.com (W.Z.); 2 Institute of Animal Science, Ningxia Academy of Agricultural and Forestry Sciences, Yinchuan 750002, China; maqing1973@126.com (Q.M.); wangjin8208@163.com (J.W.); zhaoweibing2008@163.com (Z.Z.)

**Keywords:** Chinese Tan sheep, birth wool length, birth weight, head coat color, genome-wide association study, *MC1R*

## Abstract

The Chinese Tan sheep is a unique breed of sheep that is typical throughout China, mainly used for fur and meat production. They are widely distributed in northwestern China and are famous for their lambskin and shiny white curly wool. In this study, the phenotypic traits of wool length, birth weight, and head coat color were evaluated in 256 Chinese Tan sheep breeds. Whole genome sequencing generated 23.67 million high-quality SNPs for genome-wide association studies (GWAS). We identified 208 significant SNPs associated with birth wool length, implicating *RAD50*, *MACROD2*, *SAMD5*, *SASH1*, and *SPTLC3* as potential candidate genes for this trait. For birth weight, 1056 significant SNPs, with 76.89% of them located on chromosome 2, were identified by GWAS, and *XPA*, *INVS*, *LOC121818504*, *GABBR2*, *LOC101114941*, and *LOC106990096* were identified as potential candidate genes for birth weight. The GWAS for head coat color identified 1424 significant SNPs across three chromosomes, with 99.65% on chromosome 14, and *SPIRE2*, *TCF25*, and *MC1R* as candidate genes were found to be possibly involved in the development of the black-headed coat color in sheep. Furthermore, we selected head coat color as a representative trait and performed an independent test of our GWAS findings through multiplex PCR SNP genotyping. The findings validated five mutation sites in chromosome 14 (14,251,947 T>A, 14,252,090 G>A, 14,252,158 C>T, 14,252,329 T>G, and 14,252,464 C>T) within the exon1 of the *MC1R* gene (517 bp), as identified by GWAS in an additional 102 Tan sheep individuals, and revealed that black-headed sheep predominantly exhibited heterozygous genotypes, possibly contributing to their color change. Our results provide a valuable foundation for further study of these three economically important traits, and enhance our understanding of genetic structure and variation in Chinese Tan sheep.

## Simple Summary

The Chinese Tan sheep is an important livestock species in North China, valued for its meat, wool, and skin. Despite some progress in identifying genetic loci associated with various sheep phenotypes, understanding of the genetic bases for economically important traits such as head coat color, birth weight, and wool length has been limited. Our study leverages whole-genome resequencing data from 256 individuals to perform genome-wide association studies (GWAS), uncovering significant SNPs and their associated genes. Our research identifies some key genes influencing the aforementioned traits. Notably, we also confirm the significant association of the *MC1R* gene with head coat color. Our findings will contribute to future research in this area and the breeding of economically significant traits in sheep.

## 1. Introduction

The Chinese Tan sheep is a unique and prized breed, widely distributed across northwestern China, known for its dual utility in fur and meat production. Celebrated for their robust adaptation to harsh, dry, cold, and windy conditions, these sheep exhibit a remarkable ability to thrive in unfavorable environments [1]. Their coat coloration is particularly distinctive, featuring a predominantly solid white body with the head, neck, and face adorned by black or brown markings, creating a striking contrast. Generally speaking, lambs with large birth weight are more likely to grow healthily, and longer hair length at birth indicates better hair follicle development. Thus, identifying the genomic regions and genetic determinants associated with these characteristics (birth weight, birth wool length, and head coat color) is therefore of significant importance in the context of the Tan sheep breed.

The birth wool length, birth weight, and coat color of sheep are important economic traits that have a significant impact on breeding and production [2,3,4,5,6,7]. In recent years, with the development of genomics technology, genome-wide association studies (GWAS) have become an important tool for identifying genetic variations affecting these traits [5]. Several studies showing DNA polymorphisms and genes linked to body weight have proven vital for livestock breeding, such as *IGF-I* [8], *MSTN* [9], and *ADRB3* [10]. Regarding wool length, multiple genes and gene families (such as *KRTCAP3*, *KRTAP9*, the *KAP* family, *UBE2E3*, *RHPN2*, and *TRIM2*) are primarily involved in keratin synthesis and are thought to play a role in influencing the wool parameters with the most economic value, such as fiber diameter, wool length, and greasy wool weight [2,6,11,12,13]. Coat color is complex and affected by the interaction of multiple genes, such as *MC1R*, *ASIP*, *TYRP1*, *KIT*, and *MITF*, all of which are pivotal in the formation of coat color [7,14,15,16,17,18]. Variations within these genes can affect melanin production, melanogenesis, cellular differentiation, and migration, thereby ultimately influencing the intensity, shade, and patterning of wool pigmentation [14]. Chinese Tan sheep are famous for their lambskin and shiny white curly wool. Because most of the existing investigations into these sheep have involved a limited number of molecular markers, previous studies remain insufficient in terms of assessing the genetic influences behind numerous economically important traits. Therefore, it is necessary for us to further explore the genes associated with the control of these three complex traits in Chinese Tan sheep.

Resequencing outperforms genotyping arrays by offering broader genomic insights and higher resolution, crucially uncovering novel genetic markers for non-model species [19]. In this study, we re-sequenced a total of 256 Chinese Tan sheep genomes with a mean depth of 13.66× and identified a number of genes potentially associated with birth wool length, birth weight, and coat color via GWAS. Additionally, we employed PCR SNP genotyping to validate the associated results. These data provide a valuable genomic resource for facilitating future molecular-guided breeding and genetic improvement of domestic sheep.

## 2. Materials and Methods

All procedures involving experimental animals were approved by the Animal Welfare Committee of Ningxia University (NXU 2024-060). All efforts were made to reduce the suffering and discomfort of experimental animals.

### 2.1. Animal Selection and Phenotypic Data Collection

In this study, a total of 256 Tan sheep were included in the study population, selected from the Yanchi Tan Sheep Breeding Farm (Wuzhong, China). The selected test population had accurate production records and individual trait information (birth wool length, birth weight, and head coat color etc.), where the dates were clustered in 2023. Three production traits were primarily analyzed: birth wool length, birth weight, and head coat color. The data were all provided by the Chinese Tan sheep Breeding Farm (Yinchuan, China). All sheep were a typical breed of Tan sheep, comprising 88 black-headed sheep (male: n = 18, female: n = 70) and 168 white sheep (male: n = 39, female: n = 129). The birth wool lengths were measured on the left scapula, and the birth weights were measured for each individual (Supplementary Table S1).

### 2.2. DNA Extraction and Sequencing

Blood was collected from the jugular vein of a selected group of Tan sheep on the 35th day post-partum, and genomic DNA was extracted following the standard phenol-chloroform extraction procedure. The purity of DNA was evaluated using a NanoDropTM One UV-Vis spectrophotometer (Thermo Fisher Scientific, Waltham, MA, USA), with an OD260/280 ratio ranging from 1.8 to 2.0 and an OD260/230 ratio between 2.0 and 2.2. For genome sequencing, at least 0.5 μg of genomic DNA from each sample was used to construct a library with an insert size of ~ 300 bp on DNBSEQ-T7 (BGI, Shenzhen, China).

### 2.3. Sequence Reads Mapping and Variants Identification

The paired-end reads were mapped onto the sheep reference genome ARS-UI Ramb v2.0 (https://www.ncbi.nlm.nih.gov/assembly/GCF_016772045.1/, accessed on 1 March 2024) using the Burrows-Wheeler Aligner [20] (v.0.7.17-r1188) with the default parameters. Mapping results are listed in Supplementary Table S1. Duplicate reads were removed using SAMtools [21] (v.1.13). If multiple read pairs had identical external coordinates, only the pair with the highest mapping quality was retained. SNP calling was performed using GATK [22] (v.4.0), and the variants were further filtered using VCFtools [23] (v.0.1.16). Only biallelic SNPs with missing rates < 0.1 and minor allele frequencies (MAF) > 0.05 were included for further analysis as high-quality SNPs. All the high-quality SNPs were annotated using the ANNOVAR [24] (v.2013-06-21) and then categorized as variations in 3′-UTR, 5′-UTR, downstream, upstream, exonic, intergenic, intronic, ncRNA exonic, ncRNA intronic, ncRNA splicing, and splicing sites.

### 2.4. Population Genetics Analysis

To detect the potential substructure of the Chinese Tan sheep population in this study, principal component analysis (PCA) of high-quality SNPs for all 256 individuals was performed with the GCTA (v.1.26.0) [25]. The genetic relationships were analyzed after converting the file, including biallelic SNPs into ”PLINK PED” format with PLINK [26] (v.1.90b6.21). To characterize the genome-wide linkage disequilibrium (LD) in this population, r2 was calculated using PopLDdecay [27] (v.3.4.0) based on all high-quality SNPs.

### 2.5. GWAS

Genome-wide association analyses of the birth wool length, birth weight, and head coat color were performed using the mixed linear model (MLM) in the GCTA with gender information as a covariate. The effect of population stratification was corrected by adjusting the first three principal components as derived from the high-quality SNPs, and the proportion of variance explained by the markers was calculated using GCTA. For all high-quality SNPs, we set the thresholds as −log10 (*p*-value)  =  5 for the three traits. Furthermore, the Manhattan and the quantile–quantile (Q–Q) plots of distinct traits were drawn in R using the CMplot package [28]. LD analysis between the most significant SNPs and other SNPs in the candidate region was conducted using LDBlockShow [27], and then a GWAS locus zoom graph was generated.

### 2.6. Identification of Candidate Genes and Functional Enrichment Analysis

According to genome annotation, a gene was assumed to be a candidate associated gene if it overlapped with at least one significant SNP. To obtain an in-depth view of the biological significance of the candidate associated genes in each trait, we conducted Gene Ontology (GO, https://www.geneontology.org/, accessed on 13 March 2023) and Kyoto Encyclopedia of Genes and Genomes (KEGG, https://www.genome.jp/kegg/, accessed on 13 March 2023) pathway analyses using clusterProfiler v4.0 [29].

### 2.7. Validation Test of GWAS Findings for Head Coat Color Traits

The SNP genotyping work was performed using the imLDR™ multiplex SNP genotyping method developed by Genesky Biotechnologies Inc. (Shanghai, China). The genomics DNA, sourced from additional 102 female sheep blood samples (Supplementary Table S2), was extracted using the imLDR™ method for the simultaneous genotyping of five SNP loci within the *MC1R* gene on chromosome 14 (specifically at positions 4,251,947, 14,252,090, 14,252,158, 14,252,329 and 14,252,464) in a single ligation reaction, following the established experimental protocol [30]. The specific PCR primers used for genotyping, as well as allele details, are summarized in Supplementary Table S3.

## 3. Results

### 3.1. Summary Information of Phenotypic Data and Genotypic Data

**Figure 1:**
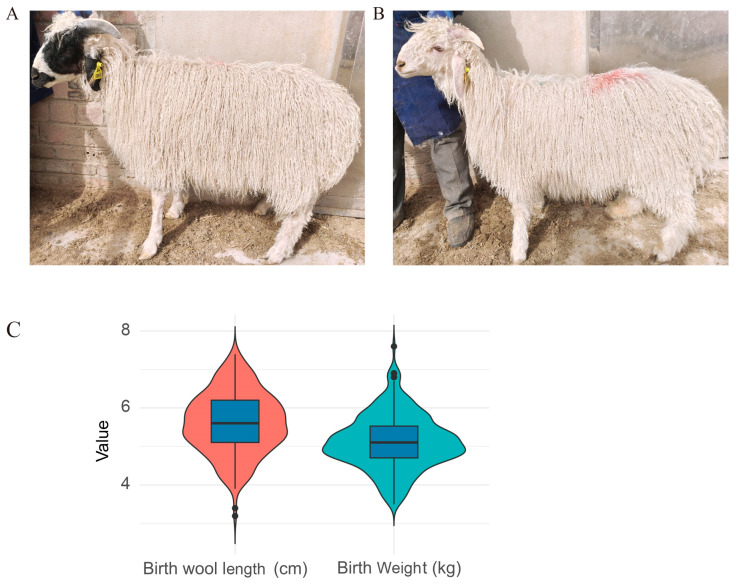
Phenotypic data and SNP type distribution in Chinese Tan sheep. (**A**) Black-headed and white Tan sheep from the Chinese population. (**B**) Violin plot showing the distribution of birth wool length and birth weight across all samples. (**C**) Sample numbers with different head coat color.

The phenotypic characteristics and sequencing data of the Tan sheep population are summarized in Table 1. The mean birth weight of the sheep was 5.11 kg, with a standard deviation (SD) of 0.71 kg, indicating a moderate variation in body mass among individuals. The mean birth wool length was 5.61 cm (SD = 0.80 cm). These physical traits highlight the diversity of phenotypic characteristics in the population. Among the 256 sampled sheep, 88 had black patterns on their heads, as shown in Figure 1A, whereas the other 168 were completely white, as shown in Figure 1B. All statistics for both traits are plotted as a violin plot and box plot in Figure 1C.

**Figure 2:**
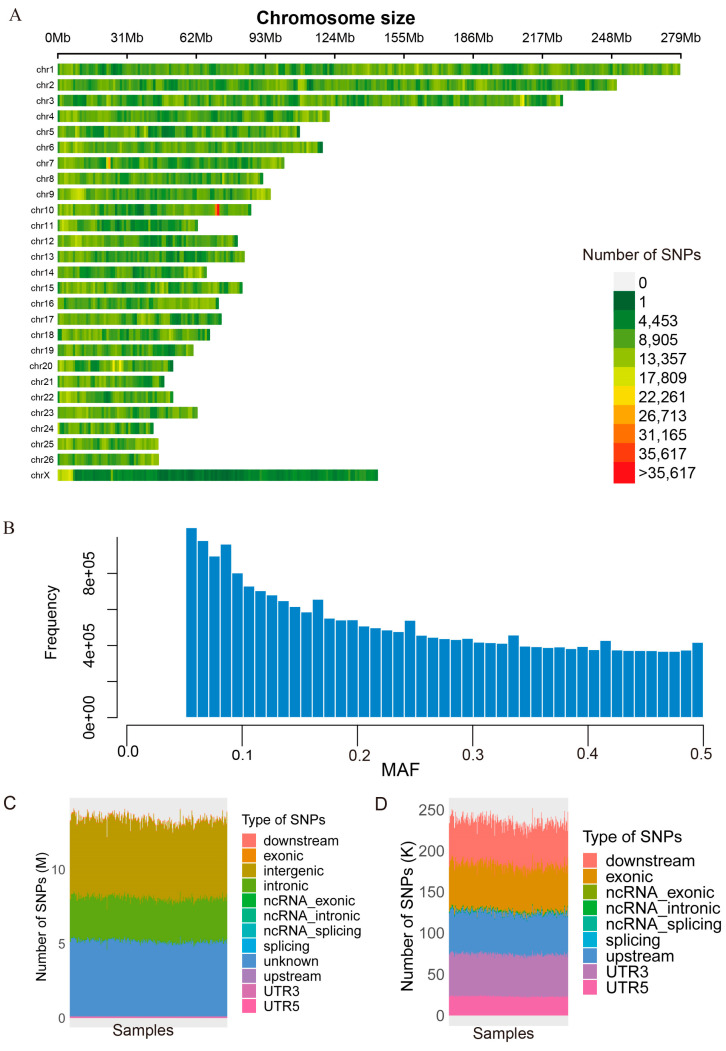
SNPs distribution in Chinese Tan sheep. (**A**) Distribution of SNPs in chromosome. (**B**) Minor allele frequency (MAF) spectrum of the identified SNPs. (**C**) Total number of SNPs (in millions) identified in different genomic regions, (**D**) The count of SNPs for types other than the top three. Each bar represents each individual samples.

For all 256 individuals, the mean sequencing depth was ~13.66× (Table 1 and Supplementary Table S1). After strict quality control, over 23.67 million high-quality SNPs were finally obtained, which were uniformly distributed across the 27 pairs of chromosomes of the sheep (Figure 2A). The site frequency spectrum of the high-quality SNPs was constructed (Figure 2B). This spectrum demonstrates an L-shaped distribution, indicating that as the MAF increases, the corresponding number of sites decreases. After annotation analysis on these high-quality SNPs across all 256 individuals, we identified that the three most prevalent types of SNPs are intergenic (with a median number of 5.26), unknown (median number of 5.05 M), and intronic (median number of 2.90 M), as shown in Figure 2C.

### 3.2. Population Structure and Linkage Disequilibrium

**Figure 3:**
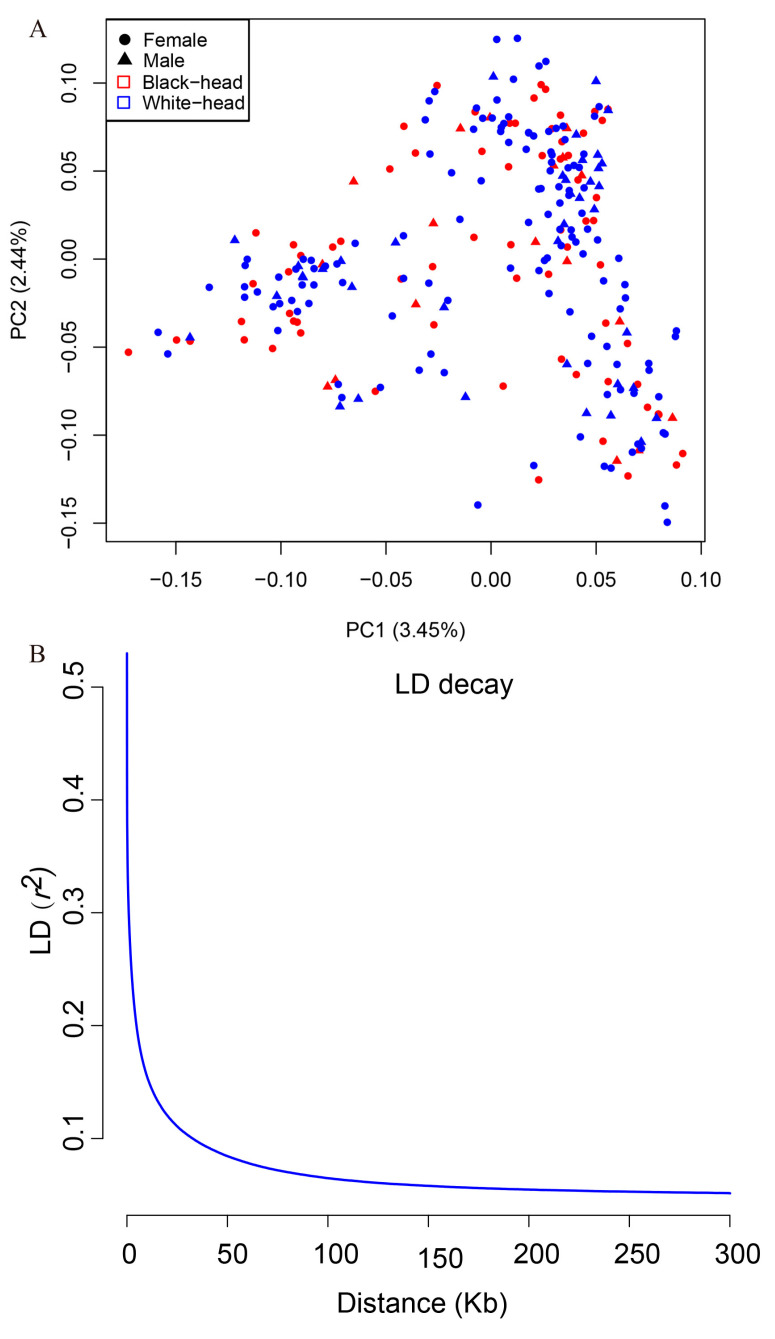
Population structure and linkage disequilibrium (LD) decay. (**A**) Principal component analysis (PCA) of Tan sheep used. (**B**) Linkage disequilibrium (LD) decay curve indicating the genomic regions under selection pressure.

To understand the population structure of the Chinese Tan sheep, we conducted principal component analysis and did not identify any adverse substructures (Figure 3A). As an indigenous breed with a long history of local cultivation, the population exhibits a history of free interbreeding among individuals of different head coat color, resulting in no discernible subpopulation structures. Linkage disequilibrium (LD, measured as r^2^) decreased to 0.2 at 4.76 kb in Chinese Tan sheep, as shown in Figure 3B. The decay of LD within domestic populations implies that artificial selection and genetic isolation have shaped their genetic architecture.

### 3.3. GWAS Results of Birth Wool Length

**Figure 4:**
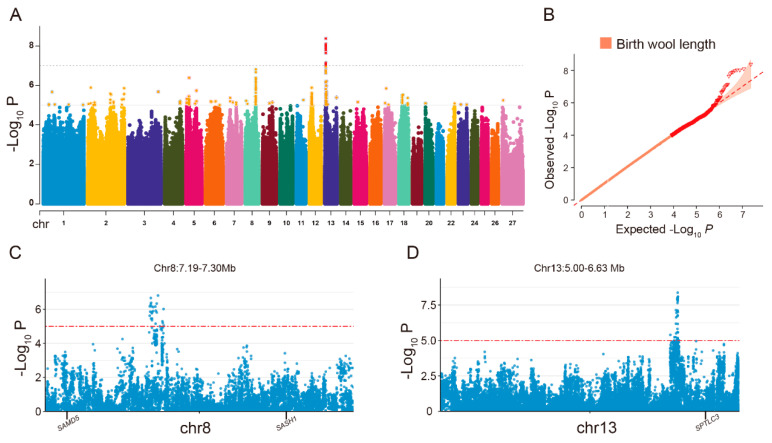
Genome-wide association study (GWAS) results for birth wool length. (**A**) Manhattan plot illustrating significant SNPs associated with birth wool length. The solid line represents a significant locus and the dashed line represents an extremely significant locus. (**B**) Q–Q plot for birth wool length. (**C**) The most significant SNPs distribution region on chromosome 8. (**D**) The most significant SNPs distribution region on chromosome 13.

In the investigation of birth wool length, GWAS revealed 208 significant SNPs that are distributed across 19 chromosomes. Notably, the highest frequency of these SNPs was detected on chromosome 12 (45 SNPs), followed closely by chromosome 13 (44 SNPs), and then chromosome 8 (30 SNPs) (Figure 4A and Supplementary Table S4). The Q–Q plot indicated that the majority of SNPs followed the expected normal distribution, while a minority exhibited significant deviations, as illustrated in Figure 4B. After annotating these genetic variations, a total of 67 SNPs were annotated in 32 genes. It is particularly striking that all of these SNPs are located within the intronic regions of the genes (Supplementary Table S4). Among the 67 SNPs, the two most significant SNPs, Chr5: 19,697,541 A>G (*p*-value = 4.11 × 10^−7^) and Chr13: 8,910,582 G>A (*p*-value = 6.22 × 10^−7^), were annotated to the *RAD50* and *MACROD2* genes, respectively. However, the majority of the most significant association signals were predominantly identified in intergenic regions, particularly in two distinct regions (Figure 4C,D). One region located on chromosome 8 (Figure 4C), spanning from positions 72,272,389 to 72,322,633, contains 24 SNPs, accounting for 80% of all significant SNPs in this chromosome. This area is adjacent to the *SAMD5* gene (approximately 271 kb away) and the *SASH1* gene (approximately 292 kb away). Another region is located on chromosome 13 (Figure 4D), with specific positions ranging from 6,250,275 to 6,296,756, near the *SPTLC3* gene (approximately 120 kb away), and this area includes 30 SNPs, representing 68.18% of all significant SNPs on this chromosome. These findings suggest that noncoding regions may be involved in the regulation of birth wool length.

### 3.4. GWAS Results of Birth Weight

**Figure 5:**
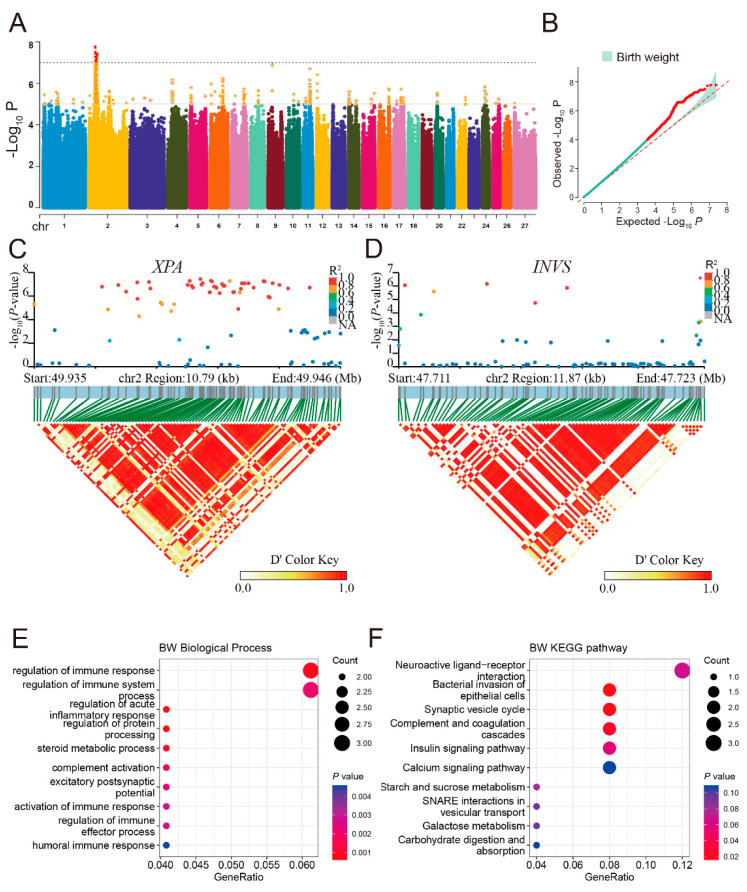
Genome-wide association study (GWAS) results for birth weight. (**A**) Manhattan plot illustrating significant SNPs associated with birth weight. The solid line represents a significant locus and the dashed line represents an extremely significant locus. (**B**) Q–Q plot for birth weight. (**C**) LD block for significant SNPs in the XPA gene region. (**D**) LD block for significant SNPs in the INVS gene region. (**E**) The dot plot shows the top 10 enriched GO terms related to biological process associated with birth weight. (**F**) The dot plot shows the top 10 enriched KEGG pathways for genes associated with birth weight.

In the study of the birth weight trait, GWAS identified 1056 significant SNPs distributed across 21 chromosomes, with 812 (76.89%) of them located on chromosome 2 (Figure 5A). The Q–Q plot indicated that the majority of SNPs followed the expected normal distribution, while a minority exhibited significant deviations, as illustrated in Figure 5B. After gene annotation, 330 SNPs were enriched in 75 genes. Notably, the most significant SNPs were found on chromosome 2, encompassing 53 SNPs linked to the *XPA* gene, 54 SNPs linked to the *INVS* gene, 18 linked to the *GABBR2* gene, and 2 SNPs linked to the *LOC121818504* gene (Supplementary Table S5). Among these, the *XPA* and *INVS* genes harbored the highest number of significant SNPs. The SNPs at positions 49,940,972 (C>T) and 49,940,978 (G>C) of chromosome 2, each with a *p*-value of 3.65 × 10^−8^, stand out as the most significant genetic variants associated with the *XPA* gene. Further analysis using the LDBlockShow tool identified haplotype blocks surrounding these two genes and revealed significant linkage disequilibrium within these regions (Figure 5C,D). Furthermore, we identified a homozygous mutation at Chr2: 54,959,878 (G>C) within the fourth exon of the *LOC121818504* gene, resulting in a non-synonymous substitution (p.T1135S). This gene is intimately associated with the process of spermatogenesis. In addition, GO and KEGG enrichment analyses of all 75 genes associated with birth weight of sheep were performed, and the results showed that two genes, *LOC101114941* and *LOC106990096*, were significantly enriched in complement and coagulation cascades (ko04610) and regulation of the immune response (GO:0050776) (Figure 5E,F), suggesting the genes associated with immunity may be involved in the regulation of birth weight.

### 3.5. GWAS Results of Head Coat Color and Validations by Multiplex PCR SNP Genotyping

**Figure 6:**
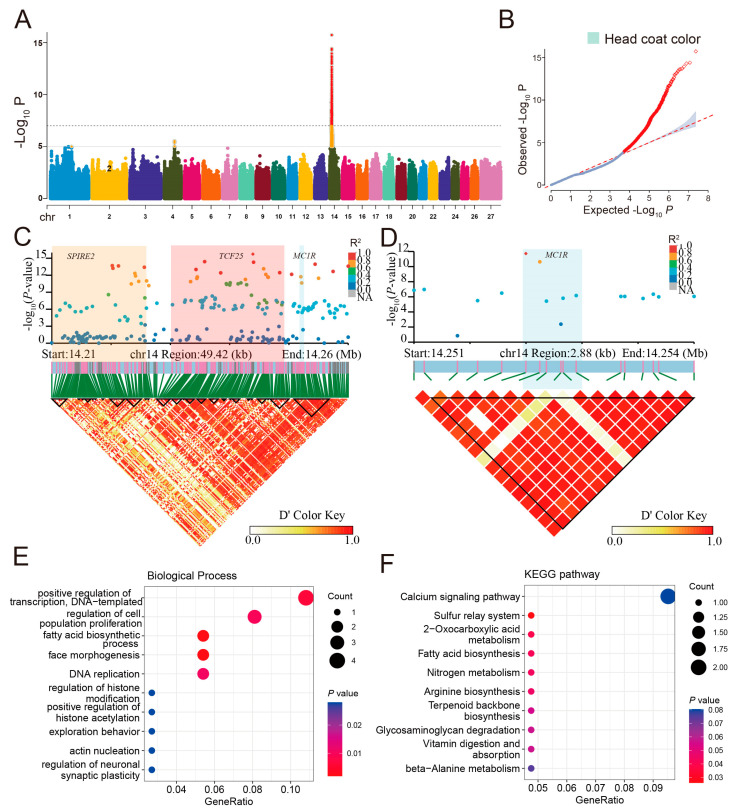
Genome-wide association study (GWAS) results for head coat color. (**A**) Manhattan plot illustrating significant SNPs associated with head coat color. The solid line represents a significant locus and the dashed line represents an extremely significant locus. (**B**) Q–Q plot for the color of black-headed sheep. (**C**) LD block for the most significant SNPs in the *SPIRE2*, *TCF25*, and *MC1R* gene regions. The shaded marker represents the gene body regions. (**D**) LD block for significant SNPs in the *MC1R* gene region. (**E**) The dot plot shows the top 10 enriched GO terms related to biological processes associated with head coat color. (**F**) The dot plot shows the top 10 enriched KEGG pathways for genes associated with head coat color.

In the study of birth head coat color, GWAS identified 1424 significant SNPs distributed across three chromosomes, with 1419 (99.65%) of them located on chromosome 14 (Figure 6A). The Q–Q plot of the head coat color trait demonstrates the presence of loci significantly associated with the trait in Figure 6B. After gene annotation, 795 SNPs were enriched in 65 candidate genes (Supplementary Table S5). A cluster of genes on chromosome 14, including *SPIRE2*, *TCF25*, and *MC1R*, were associated with the most significant SNPs (Figure 6C). Furthermore, we focused on the SNPs annotated in the coding regions and found that the *SPIRE2* gene has three synonymous SNPs, the *TCF25* gene has six synonymous SNPs, and the *MC1R* gene contains two non-synonymous and three synonymous SNPs located in exon 1 (Table 2). It is particularly noteworthy that the *MC1R* gene is known as a regulator of eumelanin and phaeomelanin production in the melanocyte [15,17,31], and our results concurred with previous research [15], suggesting that these mutation sites on the *MC1R* gene may be closely related to head coat color changes (Figure 6D).

We employed LDBlockShow to identify haplotype blocks surrounding the three genes and the results showed part of the *SPIRE2* and the entirety of the *TCF25* and *MC1R* genes exhibited strong linkage disequilibrium (Figure 6C,D). In addition, through GO enrichment analysis of 65 candidate genes associated with head coat color of sheep, we identified some significantly related terms (Figure 6E), such as fatty acid biosynthetic processes (GO:0006633) and the regulation of cell population proliferation (GO:0042127). KEGG enrichment analysis revealed significant gene enrichment in the sulfur relay system, 2-oxocarboxylic acid metabolism, fatty acid biosynthesis, nitrogen metabolism and, arginine biosynthesis (Figure 6F). Candidate genes *ACSF3*, *MLYCD*, *BRD7*, *FANCA*, *OSGIN1*, *CTU2*, *GPT2*, and *CA5A* were identified to be involved in these go terms and function pathways, suggesting their roles in controlling the head coat color through the regulation of metabolism.

**Figure 7:**
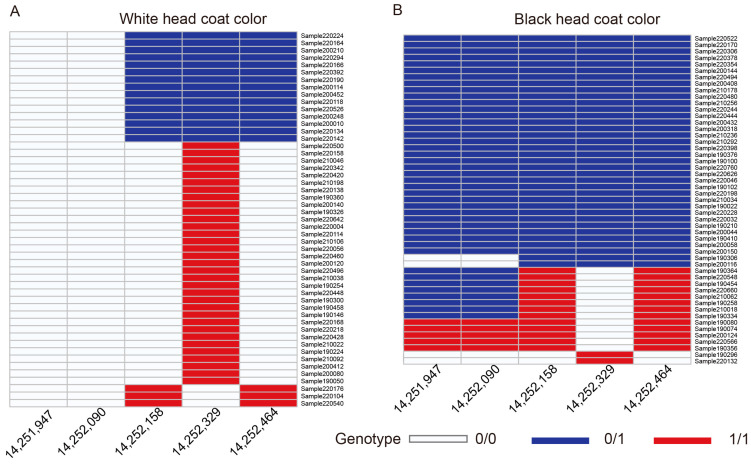
Independent validation of five candidate SNPs associated with head coat color. (**A**) Genotyping results of 51 sheep with black wool color. (**B**) Genotyping results of 51 sheep with white wool color. White box in heatmap indicates the 0/0 genotype, with no mutation; blue indicates the 0/1 genotype, with heterozygous mutation; red indicates the 1/1 genotype, with homozygous mutation. The numbers below the heatmap represent the SNPs located at different positions on chromosome 2.

For the independent verification test for our GWAS analysis, we designed five pairs of primers for five SNPs significantly associated with the head coat color trait of Tan sheep (Supplementary Table S2). Fortunately, these primers successfully amplified the expected target fragments. Subsequently, we used these five pairs of primers to perform PCR amplification on blood DNA samples from 102 additional Tan sheep individuals. The results confirmed the mutation sites identified in our GWAS analysis (Figure 7 and Supplementary Table S2). Genotyping data analysis revealed that loci Chr14: 14,251,947 and Chr14: 14,252,090 predominantly presented heterozygous genotypes (T/A and G/A, respectively) in black-headed sheep, whereas homozygous genotypes (T/T and G/G) were more frequently observed in white-headed sheep (Supplementary Table S2). The other three SNPs are primarily found in the heterozygous state in black-headed sheep (accounting for approximately 70.5%), and are also present in some small number of white sheep (accounting for approximately 29.4%), indicating the possibility of genetic exchange that may exist or the complexity of these sites in determining head coat color.

## 4. Discussion

This study presents a comprehensive whole-genome resequencing analysis of 256 Chinese Tan sheep, focusing on economically important traits. Our exploration of the sheep genome-wide SNPs was particularly directed towards understanding the genetic markers associated with economically important traits such as birth weight, birth wool length, and coat color. In this study, over 23.67 million SNPs were obtained from the genomes of 256 Chinese Tan sheep through high-throughput sequencing. The sequencing results significantly expanded the public SNP database and enriched our understanding of the genetic landscape of Chinese sheep.

As an important economic commodity, the quality and economic value of wool are influenced by the fineness, length, crimp, and color of the wool fiber. Breeding programs often focus on these traits to enhance the economic worth and desirability of the wool produced. Specific gene variants, such as variant D of the *KRTAP6-1* gene in Tan sheep, are correlated with longer straight fiber lengths at birth, and this effect is likely to extend into later growth phases [32,33]. In the present study, we focused on the birth wool length and identified *MACROD2* gene as a candidate gene for birth wool length in Chinese Tan sheep. The MACROD2 protein is a deacetylase involved in removing ADP-ribose from mono-ADP-ribosylated proteins [34]. Interestingly, recent research has identified that *MACROD2* is associated with the fiber diameter standard deviation [35]. Surprisingly, another recent study also found that the *MACROD2* gene is associated with the number of nipples [3,36]. These studies and our results highlight the pleiotropic effects of the *MACROD2* gene, indicating its influence on multiple traits.

Birth weight, as an early phenotypic indicator, significantly influences lamb survivability and growth performance over time [37,38]. Low birth weight lambs (<4 kg) tend to have compromised muscle development and adipose tissue growth. In comparison to their higher birth weight counterparts (>5.5 kg), these lambs exhibit reduced performance during fattening, greater fat accumulation, elevated saturated fatty acid content in their meat, and decreased meat tenderness [39]. Additionally, birth weight plays a role in the ease of lambing, which has subsequent implications for animal welfare and economic viability [40]. We investigated birth weight using GWAS and identified some candidate genes, including *XPA*, *INVS*, *LOC121818504*, and *GABBR2*. GWAS with a focus on birth weight in other animals have been conducted widely, such as in pigs [41,42], cattle [43,44], and goats [34]. However, there is a near absence of identical candidate genes across animals (*SKOR2*, *SMAD2*, *VAV3*, *NTNG1*, *AACS*, *APOB*, *OSBPL10*, and *LRP1B* in pigs; *ABCA12*, *FLRT2*, *LHX4*, *MAP3K5*, *NRAC*, *NTNG1*, *PIGN*, and *ZNF75A* in cattle; *MAPK3*, *LDB2*, and *LRP1B* in goats), a discrepancy likely attributable to polygenic effects and the varying heritability of these genes across different species. The immune system of ewes has a significant impact on the birth weight of their offspring [45]. We have identified multiple pathways related to immunity (Figure 5E); thus, we speculate that two genes, *LOC101114941* and *LOC106990096*, associated with immunity may also be potential genes associated with birth weight.

Coat color, as a qualitative trait in animals, has been the subject of extensive study, leading to the discovery of numerous genes that significantly influence this trait. A number of genes are known to dictate coat coloration in animals, with *ASIP* [4,16,17,18] and *MC1R* [15,17,31] among the most extensively studied. We employed GWAS and multiplex PCR SNP genotyping methods to confirm the significant association of the *MC1R* gene with coat color, identifying five relevant SNPs, particularly the two non-synonymous significant genetic loci (Chr14: 14,251,947, c.218 T>A, p.73 Met>Lys; Chr14: 14,252,090, c.361 G>A, p.121 Asp>Asn) that are exclusively present in black color sheep, which are all in concurrence with previous research [15], further validating the association between these genetic markers and the head coat color phenotype. Furthermore, our research breaks new ground by identifying *TCF25* and *SPIRE2* as emerging candidates implicated in the pigmentation process. Strong linkage disequilibrium blocks support our results, thereby broadening our understanding of the genetic architecture of coat color. Although some research studies have explored multiple facets of the Tan sheep, such as its meat characteristic [46], coat coloration [47], curly fleece [48], and rumen microbial composition [49], a number of economically important traits have yet to receive in-depth research attention. In this study, we leveraged whole-genome sequencing data to perform a comprehensive genome-wide association study on the birth weight and birth wool length of Chinese Tan sheep for the first time, in addition to delving into the head coat color trait, which has garnered previous research interest.

Our study emphasizes the significance of genomic diversity and how selective breeding has sculpted the genetic landscape of Chinese Tan sheep, shedding light on the effects of domestication practices. The observed patterns of genomic diversity and linkage disequilibrium decay (decreased to 0.2 at 4.76 kb) suggest that domestication and subsequent breeding practices have shaped the genetic architecture of Chinese Tan sheep. The identification of SNPs and candidate genes associated with three traits will offer valuable resources for enhancing growth performance in sheep and warrants further functional analysis.

## 5. Conclusions

In summary, our study delves significantly into the genomic landscape of Chinese Tan sheep, offering deep insights into the genetic underpinnings of key phenotypic traits like birth weight, birth wool length, and coat color. Through GWAS, we identified 208 significant SNPs for birth wool length, predominantly on chromosome 12, with *RAD50*, *MACROD2*, *SAMD5*, *SASH1*, and *SPTLC3* emerging as crucial candidate genes. For birth weight, 1056 SNPs were detected, mainly on chromosome 2, pinpointing *XPA*, *INVS*, *LOC121818504*, *GABBR2*, *LOC101114941*, and *LOC106990096* as significant. The coat color GWAS revealed 1424 SNPs, largely on chromosome 14, with *SPIRE2*, *TCF25*, and *MC1R* being identified as important candidate genes. Notably, we employed GWAS and multiplex PCR SNP genotyping methods to confirm the significant association of the *MC1R* gene with coat color, identifying five relevant SNPs, particularly the two non-synonymous significant genetic loci (Chr14: 14,251,947, c.218 T>A, p.73 Met>Lys; Chr14: 14,252,090, c.361 G>A, p.121 Asp>Asn) that are exclusively present in black color sheep.

## Figures and Tables

**Table 1:** Summary statistics of phenotypic and sequencing data in Tan Sheep population.

Item	Birth Weight (kg)	Birth Wool Length (cm)	Clean Base (G)	Depth (×)
mean	5.11	5.61	37.89	13.66
SD	0.71	0.80	15.20	5.34
min	3.50	3.20	26.20	9.56
max	7.60	7.40	221.20	77.87
CV	13.79	14.23	40.12	39.08

**Table 2:** Functional annotation of SNPs located in *MC1R*.

Chr	Start	End	Ref	Alt	*p* Value	Annotation	Type
chr14	14,251,947	14,251,947	T	A	1.8665 × 10^−12^	*MC1R*:exon1:c.T218A:p.M73K,	nonsynonymous
chr14	14,252,090	14,252,090	G	A	2.30469 × 10^−11^	*MC1R*:exon1:c.G361A:p.D121N,	nonsynonymous
chr14	14,252,158	14,252,158	C	T	3.7276 × 10^−6^	*MC1R*:exon1:c.C429T:p.Y143Y,	synonymous
chr14	14,252,329	14,252,329	T	G	1.54022 × 10^−6^	*MC1R*:exon1:c.T600G:p.L200L,	synonymous
chr14	14,252,464	14,252,464	C	T	6.79962 × 10^−7^	*MC1R*:exon1:c.C735T:p.I245I,	synonymous

## Data Availability

The raw sequence data are available from the Sequence Read Archive (SRA) database at the National Center for Biotechnology Information (NCBI) with the BioProject ID: PRJNA1124430.

## Supplementary Materials

The following supporting information can be downloaded at: https://www.mdpi.com/article/10.3390/ani14233495/s1, Table S1: Summary of phenotypic data and mapping statistics for the 256 Chinese Tan sheep that were sequenced in this stud; Table S2: Genotype information and coat color association for SNP markers in sheep; Table S3: The primer sequence information of PCR amplification; Table S4: The significant SNPs and annotated genes about birth wool length; Table S5: The significant SNPs and annotated genes about birth weight; Table S6: The significant SNPs and annotated genes about head coat color.
